# Effects of Methylmercury Contained in a Diet Mimicking the Wayana Amerindians Contamination through Fish Consumption: Mercury Accumulation, Metallothionein Induction, Gene Expression Variations, and Role of the Chemokine CCL2

**DOI:** 10.3390/ijms13067710

**Published:** 2012-06-21

**Authors:** Jean-Paul Bourdineaud, Muriel Laclau, Régine Maury-Brachet, Patrice Gonzalez, Magalie Baudrimont, Nathalie Mesmer-Dudons, Masatake Fujimura, Aline Marighetto, David Godefroy, William Rostène, Daniel Brèthes

**Affiliations:** 1 Bordeaux University-CNRS, UMR EPOC 5805, Arcachon Marine Station, Place du Docteur Peyneau, Arcachon, 33120, France; E-Mails: m.laclau@epoc.u-bordeaux1.fr (M.L.); r.maury-brachet@epoc.u-bordeaux1.fr (R.M.-B.); p.gonzalez@epoc.u-bordeaux1.fr (P.G.); m.baudrimont@epoc.u-bordeaux1.fr (M.B.); n.mesmer-dudons@epoc.u-bordeaux1.fr (N.M.-D.); 2 National Institute for Minamata Disease, Pathology Section, Department of Basic Medical Sciences, 4058-18 Hama, Minamata, Kumamoto 867-0008, Japan; E-Mail: fujimura@nimd.go.jp; 3 Laboratoire de Neurosciences Cognitives, Bordeaux University-CNRS UMR 5106, Avenue des Facultés, Talence, 33405, France; E-Mail: a.marighetto@cnic.u-bordeaux1.fr; 4 Institut de la Vision, UMRS 968, INSERM-University Pierre et Marie Curie, 17 Rue Moreau, Paris, 75012, France; E-Mails: david.godefroy@inserm.fr (D.G.); william.rostene@inserm.fr (W.R.); 5 Institut de Biochimie et Génétique Cellulaires, UMR 5095 CNRS-Bordeaux University, 1 rue Camille Saint-Saëns, Bordeaux, 33077 Cedex, France; E-Mail: daniel.brethes@ibgc.cnrs.fr

**Keywords:** methylmercury, fish consumption, chemokine, mercury accumulation, metallothionein, demethylation

## Abstract

Methylmercury (MeHg) is a potent neurotoxin, and human beings are mainly exposed to this pollutant through fish consumption. We addressed the question of whether a diet mimicking the fish consumption of Wayanas Amerindians from French Guiana could result in observable adverse effects in mice. Wayanas adult men are subjected to a mean mercurial dose of 7 g Hg/week/kg of body weight. We decided to supplement a vegetarian-based mice diet with 0.1% of lyophilized *Hoplias aimara* fish, which Wayanas are fond of and equivalent to the same dose as that afflicting the Wayanas Amerindians. Total mercury contents were 1.4 ± 0.2 and 5.4 ± 0.5 ng Hg/g of food pellets for the control and aimara diets, respectively. After 14 months of exposure, the body parts and tissues displaying the highest mercury concentration on a dry weight (dw) basis were hair (733 ng/g) and kidney (511 ng/g), followed by the liver (77 ng/g). Surprisingly, despite the fact that MeHg is a neurotoxic compound, the brain accumulated low levels of mercury (35 ng/g in the cortex). The metallothionein (MT) protein concentration only increased in those tissues (kidney, muscles) in which MeHg demethylation had occurred. This can be taken as a molecular sign of divalent mercurial contamination since only Hg^2+^ has been reported yet to induce MT accumulation in contaminated tissues. The suppression of the synthesis of the chemokine CCL2 in the corresponding knockout (KO) mice resulted in important changes in gene expression patterns in the liver and brain. After three months of exposure to an aimara-containing diet, eight of 10 genes selected (*Sdhb*, *Cytb*, *Cox1*, *Sod1*, *Sod2*, *Mt2*, *Mdr1a* and *Bax*) were repressed in wild-type mice liver whereas none presented a differential expression in KO *Ccl2*^−/−^ mice. In the wild-type mice brain, six of 12 genes selected (*Cytb*, *Cox1*, *Sod1*, *Sod2*, *Mdr1a* and *Bax*) presented a stimulated expression, whereas all remained at the basal level of expression in KO *Ccl2*^−/−^ mice. In the liver of aimara-fed mice, histological alterations were observed for an accumulated mercury concentration as low as 32 ng/g, dw, and metal deposits were observed within the cytoplasm of hepatic cells.

## 1. Introduction

Methylmercury (MeHg), the more toxic form of mercury by ingestion, is a potent neurotoxin, and human beings are mainly exposed to this pollutant through fish consumption, although under certain conditions dermal contact and inhalation of mercury vapor are the main exposure routes (such as in the case of industrial workers or artisanal gold miners). Two large studies among others, contradictory in their conclusions, have been performed in recent years to assess the impact of exposure to MeHg through fish consumption on the fetal brain in the Faroe Islands [1] and in the Seychelles [2]. These studies focused on the possible consequences of prenatal MeHg exposure, but only a few studies aim to understand the impact of mercury-enriched fish consumption on adult populations. In the Amazonian basin, Amerindian populations are exposed to MeHg through consumption of fish containing biomagnified concentrations of this pollutant partly coming from gold mining activities. Amerindian children from the upper Maroni in French Guiana were highly contaminated with a mean of 12 μg Hg/g in hair, and were afflicted by neurological disorders, such as poor coordination of the legs, and decreased performance in the Stanford-Binet copying score [3,4]. However, contradictory data have been collected linking mercurial contaminations and their impact on Amazonian populations’ health. The Mundurukus Amerindians (Pará state, Brazil) present elevated mean levels of mercury in hair (14 to 16 μg/g) without overt mercury intoxication signs [5]. The same conclusions could be drawn from the communities of Brasília Legal and São Luís do Tapajós (Pará state, Brazil) [6]. In contrast, other authors established a correlation between mercury hair content and impact on motor performance and visual capability of a riverine population of the Tapajós river (Pará state, Brazil) [7–10]. Since the epidemiological studies so far available are contradictory, we found it necessary to experiment on adult animals to model the possible effects of mercurial contamination through fish consumption.

In the time-course of a study dedicated to mercurial contaminations in French Guiana, we prepared mice diets by adding mercury-containing flesh from fish caught in French Guiana. The *Hoplias aimara* species, which Amerindians are fond of, was chosen because this fish is highly contaminated by MeHg (4 to 12 μg/g dw), and because this single species represents 27% of the Wayanas’ dietary mercury intake and 10.7% of the total flesh they consume [11]. Four diets containing 0, 0.1, 1, and 7.5% aimara flesh, representing 0, 5, 62, and 520 ng MeHg per g, respectively, were given to four groups of mice for a month. The lowest fish regimen led to a mercurial contamination pressure of 1 ng mercury per day per g of body weight, which is precisely that afflicting the Wayana Amerindians. The expression of several genes was modified with mercury intoxication in the liver, kidney, and hippocampus, even at the lowest tested fish regimen. In the muscles of mice fed the lowest fish-containing diet, cytochrome *c* oxidase activity decreased to 45% of that of the control muscles [12]. Since the 0.1% fish-containing regimen proved to affect gene expression and muscle mitochondrial respiration after just one month of exposure, we decided to carry out an experiment with such a contamination pressure exposing mice for 19 months in order to check whether the adverse affects worsen with time. Two main differences can be pinpointed between the present proposed study and almost all of those dealing with MeHg toxicity published up to now in the literature: first, the form of MeHg used, naturally associated to fish in the former and added pure in the latter; second, the unprecedented low dose of MeHg with levels one hundred-times below those used in the published articles.

MeHg affects brain development and results in neuroinflammatory and neurotoxic effects, and MeHg toxicity is mediated by the generation of reactive oxygen species leading to the induction of target proteins and among others cytokines [13]. The chemokine CCL2 is suggested to have a neuromodulatory effect and to play an essential role in various neuroinflammatory processes [14,15]. Using *in vitro* experiments on pure rat cortical neurons in culture, we observed by blockade of the CCL2/CCR2 neurotransmission an increased neuronal cell death in response to MeHg neurotoxicity. Furthermore, CCL2 could blunt *in vitro* the decrease in glutathione levels induced by MeHg [16]. The second objective of this study was therefore to test the possibility that the chemokine CCL2 could oppose the deleterious effects of MeHg using knockout CCL2 mice, and to look at the interplay between MeHg and CCL2 deficiency at the level of gene expression.

After 19 months feeding with a diet containing lyophilized aimara fish flesh at a final concentration of 5 ng MeHg/g of food giving a contamination pressure of 1 ng MeHg/day/g of body weight, we analyzed the mercury accumulation in tissues, the MeHg demethylation, the metallothionein content, the gene expression perturbation, and the contribution of the *Ccl2* gene to the genetic response of tissues to MeHg contamination. We also performed a histological analysis of MeHg impact on the liver.

## 2. Materials and Methods

### 2.1. Rationale Underlying the Preparation of the Mice Diets

In French Guiana, a survey of the daily mercury intake in the Wayana Amerindian population has been carried out. Adult men aged between 25 to 45 years were daily ingesting a mean of 61 g mercury [11]. Their mean body weight being around 60 kg, the mercurial contamination pressure was 1 ng Hg/day/g body weight. For mice weighing around 25 g, such a dose corresponds to a daily ingestion of 25 ng mercury brought by a mean consumption of 5 g pellets per day per animal. Therefore, to mimic the Wayanas’ contamination, mice food pellets had to contain 5 ng Hg/g brought by dry fish flesh supplementation. The *H. aimara* fish whose flesh was used was caught in French Guiana in the Sinnamary River, known to be contaminated by methylmercury mostly originating from the Petit-Saut hydroelectric reservoir [17]. Fish were frozen and stored at −20 °C before being dried by lyophilization. After blending several pieces of flesh from different animals, the dry flesh of these animals contained 3.3 to 5 μg Hg/g. Thus, a diet containing 0.1 to 0.15% of this fish flesh could mimic Wayana’s contamination. To unravel the exact contribution of mercury and fish nutriments to the observed effects, we decided to prepare, in addition to the control and aimara-containing diets, a third diet enriched with 0.1% of salmon flesh. Indeed, it is impossible to catch aimara devoid of mercury. Even the juvenile aimaras present an unacceptable mercury burden. For instance, the smallest aimara fish that was caught in French Guyana weighed 51 g, was 5 cm long, and contained 0.28 μg of mercury per g of body weight on a dry weight basis (the largest aimara weighed 10 kg, was 80 cm long, and contained 6.2 μg of mercury per g) [18]. Salmon bred in Norway aquacultures was selected because their flesh contains very low amounts of MeHg. Salmon fillets were purchased frozen in a local shop belonging to a French brand selling frozen foodstuffs (Picard). The information delivered by Picard on the fish thus sold was as follows: salmon, *Salmo salar*, was bred in Norway (reference number 017217) and its nutritional composition was 18% proteins, 14% lipids, and no carbohydrates. Fish were thawed, minced and lyophilized. Then the fish powders were ground in a kitchen blender. Before incorporation in the food pellets, the lyophilized salmon contained 98.6 ± 5 ng Hg/g of flesh, 50-times less than in lyophilized aimara flesh.

The special salmon and aimara diets have been manufactured by Special Diets Services (Witham, Essex, United Kingdom; French commercial representation: Dietex, Saint-Gratien, France). The control diet was mainly vegetarian (Rat and Mouse n°1 maintenance diet, abbreviated to RM1 diet, Special Diets Services). According to Special Diets Services, RM1 diet is made by blending wheat, barley, wheatfeed, de-hulled extracted toasted soya, soya protein concentrate, macro and micro minerals, soya oil, whey powder, amino acids, and vitamins. The nutrient composition of the lyophilized aimara flesh has already been described [12]. Those of lyophilized salmon along with that of salmon-containing diet, aimara-containing diet, and control RM1 are given in Table 1 (the analyses were carried out by Special Diets Services).

A comparison of the diet compositions showed that there were no substantial differences between the control and the salmon and aimara diets. However, the salmon diet contained 0.06% more fat than the control and aimara diets, and both the salmon and aimara diets contained 0.08% more protein than the control diet.

We quantified the total mercury content of the two prepared regimens, and found 1.5 ± 0.2 and 5.4 ± 0.5 ng Hg/g of food pellets for the salmon and aimara diets, respectively. The control RM1 diet contained 1.4 ± 0.2 ng Hg/g of food pellets. The mercury species contained in the control RM1 diet was found to be 100% inorganic which is not surprising since it is the one accumulated by plants, whereas the contribution of the methylated species to the total mercury load was found to be over 95% in salmon and in aimara flesh. Therefore, the fraction of the methylated form of mercury is 6% and 70% in the salmon and aimara diets, respectively. The content in several other metals of the lyophilized aimara flesh has already been described [12]. Those of the lyophilized salmon along with that of salmon-containing diet, aimara-containing diet, and control RM1 are given in Table 2. Metals have been assayed by ICP-MS (Antellis, Toulouse, France). The diets and fish flesh levels were below the detection threshold for Ag (<0.02 mg·kg^−1^), Au (<0.05 mg·kg^−1^), Bi (<0.02 mg·kg^−1^), Sb (<0.5 mg·kg^−1^), Sn (<0.5 mg·kg^−1^), Tl (<0.05 mg·kg^−1^), and V (<0.5 mg·kg^−1^). The RM1 control diet contained greater metal concentrations than aimara and salmon fleshes probably due to the fact that plants accumulate heavy metals from soil. Consequently, besides mercury, the salmon and aimara diets are not distinguishable from the control diet in terms of metal content.

### 2.2. Mice Treatment and Tissue Sampling

Three-week-old male mice of the C57Bl/6 Jico inbred strain (IFFA Credo, Lyon, France), or mice deficient in CCL2 (B6.129S4-Ccl2tm1Rol/J) on the same C57Bl/6 background [19] were purchased from Jackson Laboratory (Bar Harbor, ME, USA). They were socially housed in standard conditions: room temperature (23 °C), 12/12 light cycles and *ad libitum* food and water. Experiments were performed in compliance with the European Community Council directive of 24 November 1986 (8616091 EEC). At the time of sampling, mice were killed by cervical disruption followed by decapitation. Then, all the tissues were dissected for mercury quantification, microscopy, metallothionein determination and gene expression analysis. Harvested muscles were those from thighs and legs. Blood was collected in tubes containing 10 mM EDTA in order to avoid clot formation.

#### 2.2.1. Experiment 1

Two groups of wild-type C57Bl/6 mice were fed the control RM1 and aimara-containing diets, respectively, for 19 months. Mice tissue samplings were carried out after 3, 7 and 19 months to perform mercury bioaccumulation quantification, metallothionein quantification (after three and seven months), and microscope analysis after 7 months of exposure.

#### 2.2.2. Experiment 2

Three groups of wild-type C57Bl/6 mice were fed the control RM1, and aimara- and salmon-containing diets, respectively, for 14 months. Mice tissue samplings were carried out after three months to perform mercury bioaccumulation quantification, and after 14 months to perform mercury bioaccumulation quantification and gene expression analysis.

#### 2.2.3. Experiment 3

Three groups of wild-type C57Bl/6 mice were fed the control RM1, and aimara- and salmon-containing diets, respectively, for three months. In parallel, two groups of *Ccl2*-knock-out C57Bl/6 mice were fed the control RM1 and aimara-containing diets, respectively. Mice tissue samplings were carried out after three months to perform mercury bioaccumulation quantification and gene expression analysis.

### 2.3. Mercury Quantification

Total Hg concentrations in mice tissues were determined by flameless atomic absorption spectrometry. Analyses were carried out automatically after thermal decomposition at 750 °C under an oxygen flow (AMA 254, Prague, Czech Republic). The detection limit was 0.01 ng Hg. MeHg concentrations were determined in mice tissues after extraction with saturated potassium hydroxide in methanol solution (250 g/L) for 12 h in the dark at room temperature and then 3 h at 75 °C. The pH of this solution was then adjusted to 4.9 with 2 M sodium acetate before ethylation with sodium tetraethylborate (1% NaBEt_4_, for 30 min). Volatile Hg species were cleared out with mercury-free nitrogen and collected on Tenax. All mercury species were separated by isothermal gas chromatography (60 °C) prior to pyrolysis (900 °C) and detected using cold vapor atomic fluorescence spectrometry (CVAFS, Tekran detector 2500, Toronto, Ontario) [20]. The detection limit was 10 pg of Hg. The validity of the analytical methods was checked during each series of measurements using three standard biological reference materials (TORT2, DOLT2 and DOLT3); Hg values were consistently within the certified value range (data not shown). Stomach and intestines were washed from processed food and fecal matter before analysis.

### 2.4. Metallothionein Quantification

The levels of total metallothionein proteins (MT) in the kidney, liver, muscles and the brain were determined by mercury-saturation assay [21]. MT analysis was conducted on three replicates from three different mice per exposure groups, the saturation assay being repeated twice per sample. This technique is based on the quantification of Hg bound to the saturated MTs. The denaturation of non-MT proteins was performed with trichloroacetic acid and excess Hg not bound to the MTs was removed by scavenging with lyophilized beef hemoglobin (Sigma) prepared in 30 mM Tris-HCl buffer (pH 8.2 at 20 °C). The final supernatant was then quantitatively recovered and used for Hg determination by flameless atomic absorption spectrometry (AMA254, Altec, Prague, Czech Republic). The detection limit was estimated at 1 ng Hg. MT concentrations were expressed in nmol Hg bound/g tissue (wet weight).

### 2.5. Gene Expression Analysis

Total RNAs were extracted from 40 mg of fresh hippocampus, liver, kidney, and muscle tissues using the Absolutely RNA Miniprep kit (Stratagene), according to the manufacturer’s instructions. First-strand cDNA was synthesized from 5 μg total RNA using the Stratascript First-Strand DNA Synthesis kit (Stratagene). The cDNA mixture was stored at −20 °C until its use in real-time PCR reaction. The accession numbers of the 20 genes used in our study and the corresponding primer pairs have already been listed [22]. These genes and their corresponding proteins are: *b-actin*, cytoplasmic-actin; *Atp5a1*, ATP synthase, H^+^ transporting, mitochondrial F1 complex, alpha subunit, isoform 1; *Atp6*, ATP synthase subunit a (F-ATPase protein 6); *Bax*, Bcl2-associated X protein; *Cd11b* (*Itgam*), integrin alpha M or Cd11b antigen; *Cox1*, cytochrome *c* oxidase subunit I; *Cox4*, cytochrome *c* oxidase subunit IV isoform 1; *Cytb*, cytochrome b; *Gfap*, glial fibrillary acidic protein; *Gsta4*, glutathione S-transferase, alpha 4; *Hsp25* (*Hspb1*), heat shock protein 1, 27 kDa; *Mcp1* (*Ccl2*), monocyte chemoattractant protein or chemokine (C-C motif) ligand 2; *Mdr1a*, ATP-binding cassette, sub-family B (MDR/TAP), member 1A or multidrug resistance protein 1a (Abcb1a); *Mt-Nd4*, NADH dehydrogenase subunit 4; *Mt2*, metallothionein isoform 2; *Ndufs8*, NADH dehydrogenase:ubiquinone Fe-S protein 8; *Sdhb*, succinate dehydrogenase complex, subunit B, iron sulfur protein; *Sod1*, cytoplasmic superoxide dismutase; *Sod2*, mitochondrial superoxide dismutase; *Uqcrc2*, ubiquinol cytochrome *c* reductase core protein 2.

Real-time PCR reactions were performed in a LightCycler (Roche) as previously described [22]. Relative quantification of each gene expression level was normalized to the *b-actin* gene expression. The choice of this reference gene proved to be accurate since, in all tissue types considered, the means of cycle threshold (Ct, the number of cycles for which the PCR enters in the linear phase) related to this gene did not vary between control and salmon- or aimara-fed mice, and for instances the average Ct values recorded for this gene were between 26.54 and 27.8 for the brain and between 23.73 and 24.22 for the liver. The differential expression of a gene was calculated as the ratio of its expression, normalized to *b-actin* gene, in fish-contaminated condition to that in the control condition. Only the differential gene expression levels at least equal to or above 2 were considered.

Interindividual variability for each experimental condition was defined by mean ± standard deviation (*n* = 5). Due to the high variability of relative expression values between individuals, the distribution of results never proved to be normal, so that non-parametric tests should be used. Significant differential gene expression levels between control mice and fish-fed mice in the four organs were determined using first the Kruskall-Wallis ANOVA test followed by the Mann-Whitney *U*-test (*p* < 0.05).

### 2.6. Sample Preparation for Microscopy and Image Analysis

Liver pieces (2 mm thick) were sampled and immediately immersed in a fixing solution (3% glutaraldehyde buffered with 0.1 mM sodium cacodylate solution, pH 7.4; osmolarity 600 mosmol/L) for 12 h at 4 °C, then rinsed in a cacodylate buffer (0.1 mM, NaCl 2%). After dehydration, liver slices were embedded in Araldite in order to prepare different types of sections using an automatic ultra-microtome (Reichert). For optical microscopy, these semi-fine sections (1.5 μm) were stained by blue toluidine (1%) with methylene borate (1%) before analysis under a Leitz Orthoplan microscope. For electronic transmission microscopy, ultrafine sections (500–700 Å) were placed on grids and then observed under a MET TECNAI 12 Philips microscope (Bordeaux Imaging Center, University of Bordeaux 2).

The autometallography procedure was modified from a previously published one [23]. The basic mechanism consists in the formation of shells of metallic silver “nuclei” aggregating trace metals, and this process is obtained after covering the biological section with an emulsion (llford nuclear emulsion L4) and placing it in a bath of developer under a safelight. After drying in complete darkness, and covering with L4 emulsion, sections were rinsed in developer (Ultrafin Tetenal, Agfa, Köln, Germany) for 15 mn, in stop bath (1% acetic acid) for 1 mn, and in fixer (B&W fixer, AGE, Agfa) for 10 mn. The emulsion was checked before every experiment to test uniformity of silver grains, by covering a slide without sections. Metal deposits appear in black indicating the presence of silver shells around the metals [23,24].

Image acquisition and processing were performed with Meta Imaging (MetaView serie 7.0 and MetaMorph serie 7.0, Universal Imaging Corporation, Sunnyvale, CA, USA). To determine hepatic cells area, a graphic pencil was used to delimit area of interest. All the computations were carried out with the Statistica software (Statistica 9.1; StatSoft, Tulsa, OK, USA, 2009). Significant differences in distribution of hepatic cells surfaces between control and aimara-fed mice livers were determined using the Mann–Whitney *U*-test (*p* < 0.05).

## 3. Results

### 3.1. Mercury Quantification and Demethylation Patterns

When considering the Experiment 1, as soon as three months of exposure, a significant difference of mercury accumulation between control and aimara-fed mice could be observed (Table 3).

After 7 months of exposure the body parts and tissues displaying the highest mercury concentration were hair (180 ng/g) and kidney (300 ng/g, dw) followed by the liver (32 ng/g, dw). Surprisingly, despite the fact that MeHg is a neurotoxic compound, the brain accumulated low levels of mercury (12–15 ng/g, dw). After 19 months of exposure, the mercury concentrations increased slightly in several organs, except the kidney and stomach. Between 7 and 19 months of exposure, we quantified increased mercury concentrations in hair and brain (1.7-fold), in muscles (1.5-fold), and in the liver (1.3-fold).

MeHg demethylation was observed after three months of exposure in the kidney (40%) and the liver (30%) (Table 4). At this time, no demethylation was evidenced in muscles and the brain. After 7 months of exposure, demethylation had increased in the kidney and liver, reaching 70% in both organs, and was apparent in muscles (10%). The brain remained refractory to such a process.

In Experiment 2, a control fish group was added in which mice were fed a salmon flesh-containing diet. After three or 14 months of exposure, the salmon-fed mice accumulated the same concentrations of mercury than the control group (Table 5) with only two exceptions: eyes and kidney after 14 months of exposure. The aimara-fed mice accumulated much higher levels of mercury than the two other groups. As observed in Experiment 1, hair, the kidney and the liver were the main accumulators of mercury, and a net increase in mercury concentrations was noticed between three and 14 months of exposure. After 14 months of exposure the ratios of mercury concentrations in tissues from aimara-fed mice over that of salmon-fed mice were 24, 24 and 10 in hair, the kidney and liver, respectively.

The same conclusions can be drawn from the results of Experiment 3: after three months of exposure, no differences between the wild-type control and salmon-fed groups, and much higher mercury concentrations in tissues of mice fed the aimara-containing diet (Table 6).

The ratio of mercury concentrations in tissues from aimara-fed mice over that of salmon-fed mice were 19, 21 and 15 in hair, the kidney and liver, respectively. During the time-course of Experiment 3, we carefully measured the food pellet consumption and the production of feces. Since the urinary excretion of mercury is very low in rodents (below 1%) [25] we neglected it in our assessment. On a daily basis, we observed that mice were consuming 4.2 ± 0.18 g of food pellets/animal, and excreting 1.7 ± 0.1 g of feces/mouse. Feces contained after three months of exposure 19 ng Hg/g dry weight (Table 6) or 5.7 ng Hg/g “fresh” weight. Thus, the daily level of mercury excretion was 9.7 ng Hg/mouse. Since the aimara food contained 5.4 ng Hg/g of food pellets, this made a daily intake of 22.7 ng Hg/mouse. Therefore, feces retained 43% of the daily ingested mercury (9.7 ng of excreted Hg over 22.7 ng of ingested Hg), giving a trophic transfer rate of mercury equal to 57%.

### 3.2. Metallothionein Quantification

Metallothionein (MT) concentrations were quantified in tissues of mice sampled from the first experiment. After three months of exposure, the metallothionein content observed in the kidney was 40% higher in aimara-fed than in control mice (Table 7). After 7 months of exposure, these proteins were 100% and 120% more abundant in aimara-fed than in control mice in the kidney and muscles, respectively. In the liver, a significant 36% decrease of MT concentration as compared to control was seen after 7 months of exposure.

### 3.3. MeHg-Induced Gene Expression Perturbations

The expression of genes involved in the mitochondrial metabolism (*Atp5a1*, *Atp6*, *Cox1*, *Cox4*, *Cytb*, *Mt-Nd4*, *Ndusf8*, *Sdhb*, *Uqcrc2*), the response towards oxidative stress (*Hsp25*, *Sod1*, *Sod2*), the detoxification process (*Gsta4*, *Mdr1a*, *Mt2*), the apoptotic signaling (*Bax*), and brain microglial markers (*Cd11b*, *Gfap*, *Mcp1*) were assessed. Mice coming from the Experiment 3 were sampled for the gene expression study after three months of exposure to MeHg through fish consumption. In the liver, the respiratory genes *Sdhb*, *Cytb* and *Cox1*, representing the electron transfer chain complexes II, III, and IV, respectively, were up-regulated in the salmon-fed group compared to control group (2.5-, 4-, and 4.8-times, respectively) but repressed in the aimara-fed group compared to the salmon-fed group (3.8-, 5-, and 6.7-times, respectively) (Table 8). The *Sod1*, *Sod2*, *Mt2*, *Mdr1a*, and *Bax* genes also were repressed in the aimara-fed group compared to the salmon-fed group.

In the kidney, there were no differences between gene expression from mice fed the control and those fed salmon-containing diets. However, in the kidney from mice fed the aimara diet, a significant decrease in gene expression was noticed for *Mt-Nd4*, *Cox4*, and *Atp6* genes as compared to control mice. This down-regulated pattern of gene expression indicates that the mitochondrial metabolism was impacted by the aimara-containing diet.

In muscles, both fish-containing diets resulted in the down-regulation of the respiratory genes *Ndusf8* and *Sdhb*, and that of *Sod1*, *Sod2*, and *Bax* genes compared to the control mice. This indicated that MeHg was not involved but rather that some fish nutriments common to both salmon and aimara flesh were responsible for such a gene expression pattern.

In the brain, both fish-containing diets resulted in the up-regulation of the pro-inflammatory genes *Cd11b*, *Mcp1*, and *Gfap*, compared to control mice, suggesting that the fish-containing diets increased the inflammatory status in this organ. Nevertheless, in the brains of mice fed the aimara-containing diet, several genes were found up-regulated as compared to control mice. These genes were *Bax*, *Mdr1a, Sod1*, *Sod2*, *Cox1* and *Cytb*. In addition, *Cytb* and *Ndusf8* genes were up-regulated in the brains of mice fed the aimara flesh compared to those fed salmon.

Mice coming from the Experiment 2 were sampled for the gene expression study after 14 months of exposure to MeHg through fish consumption. In the liver, all of the 15 scrutinized genes were repressed in the aimara-fed group compared to the salmon-fed group (between 5 and 14-times) (Table 9), confirming the observations made after three months of exposure (Experiment 3).

In the kidney, both fish-containing diets resulted in the down-regulation of several genes involved in the mitochondrial respiration (*Ndusf8*, *Sdhb*), the response to oxidative stress (*Gsta4*, *Sod2*), and the general stress response (*Mt2*, *Hsp25*, *Mdr1a*). The influence of MeHg that was observed after 3 months of exposure in this organ had vanished after 14 months.

In muscles, contrary to the three months exposure, during which no influence of MeHg could be pinpointed, after 14 months of exposure, 13 out of 15 tested genes showed a down-regulation pattern of expression in the aimara-fed group compared to salmon-fed and control groups. These genes comprised all the 9 tested respiratory genes, the oxidative stress responsive genes *Gst4a*, *Sod1* and *Sod2*, and the stress responsive gene *Mdr1a*.

In the brain, the influence of MeHg that was observed after three months of exposure in this organ had disappeared after 14 months. Salmon flesh triggered the down-regulation of *Atp5a1*, *Hsp25* and *Mdr1a* genes.

The differential expression of genes in tissues of mice fed the aimara diet was recorded (Table 10) after having considered as non significant that of genes observed for both fish-containing diets (*i.e.*, genes for which expression varied after exposure to both salmon and aimara flesh), taking the control diet as a reference.

It appeared that except for brain of mice fed aimara flesh after a three month-long exposure, the differential expressions indicated a repression of the responsive genes. In addition, the time-course of gene response was different between tissues: the response observed after three months in the kidney and the brain vanished after 14 months, whereas a response was significant only after 14 months in muscles and conserved in the liver between three and 14 months.

### 3.4. Influence of the CCL2 Chemokine on MeHg-Induced Gene Expression Modifications

After three months of exposure, in mice knockout for *Ccl2* gene, much higher mercury concentrations were found in tissues of mice fed the aimara-containing diet compared to mice fed the basic diet (Table 6). The ratios of mercury concentrations in tissues from aimara-fed KO mice over that of KO mice fed the basic regimen were 17, 20 and 17 in hair, the kidney and liver, respectively, very close to those observed in *Ccl2*^+/+^ mice. Therefore, the lack of *Ccl2* gene did not influence the kinetic and pattern of MeHg accumulation within the mice bodies.

When looking at the interplay between MeHg and *Ccl2* knockout, no significant influence of aimara-containing diet was observed on gene expression in the liver, kidney and muscles of KO mice (Table 11).

In the brain from KO mice, only a down-regulation of the respiratory gene *Ndusf8* was noticed. Therefore, the loss of *Ccl2* gene resulted in a great upheaval of gene expression patterns, especially in the liver and brain, compared to wild-type *Ccl2*^+/+^ mice. Most spectacular was the absence, in the liver of KO mice, of the repression pattern that had been observed in *Ccl2*^+/+^ mice when animals were fed the aimara-containing diet. In the brain, the up-regulation of the pro-inflammatory genes *Cd11b* and *Gfap* that had been observed in *Ccl2*^+/+^ mice fed with both the aimara- and salmon-containing diets was seen no more in KO mice fed the aimara diet, meaning that somehow the *Ccl2* gene monitors the expression of these genes in response to fish consumption. In the brain of *Ccl2*^+/+^ mice fed the aimara-containing diet, seven genes out of 13 were found up-regulated, whereas six of them presented a basal expression, and the *Ndusf8* gene was even down-regulated in KO mice fed the aimara diet.

### 3.5. MeHg Causes Histological Alterations in Liver

**Figure 1:**
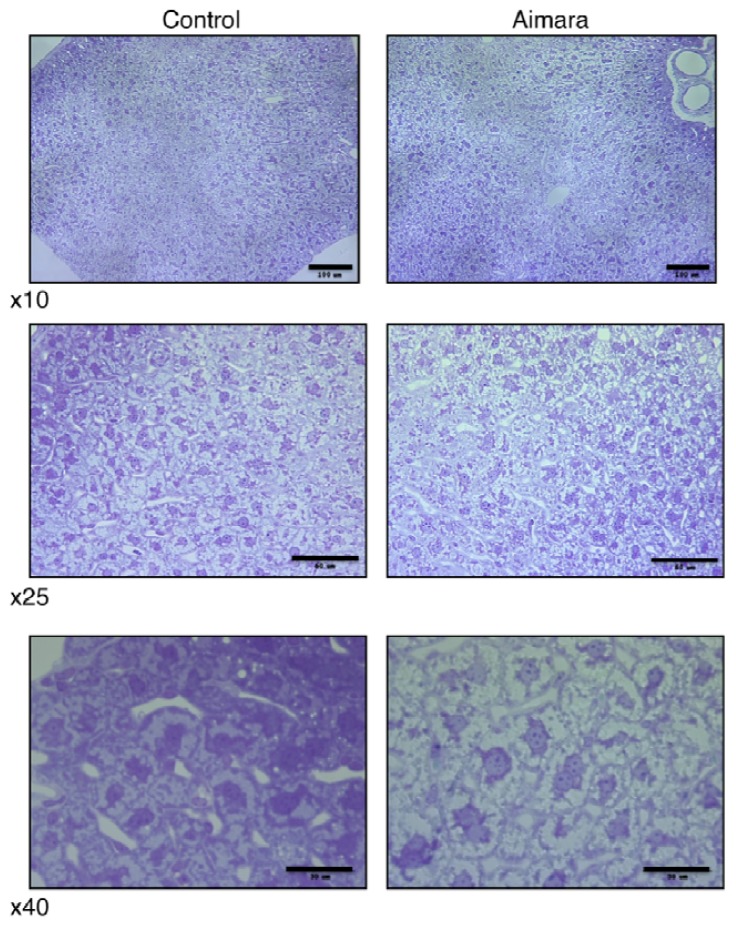
Optical microscopy of the liver in wild-type mice after 7 months of exposure to an aimara-containing diet. Scale bars are equal to 100, 60 and 30 μm from top to bottom and correspond to 10×, 25×, and 40× magnifications, respectively.

Since the organ accumulating the highest MeHg concentration was the kidney, we thought that it might be possible to observe damage at the histological level. In fact, we could see no differences between the kidneys of mice fed the control and the aimara-containing diet, either under optical microscopy or transmission electron microscopy. Surprisingly, when we looked at the liver after 7 months of exposure, we found that the area of hepatic cells tended to be larger in the livers of mice fed the aimara-containing diet (Figure 1).

When analyzing the distribution of the cell areas, we were able to highlight a shift toward the greater values, with means equal to 384 ± 13 and 461 ± 15 μm^2^ for control and aimara-fed mice, respectively (Table 12), the difference being statistically significant as assessed by the Mann-Whitney *U*-test (*p* < 0.05). 62% of the control cells had an area comprising between 100 and 400 μm^2^, against 37% for the aimara-fed mice.

**Figure 2:**
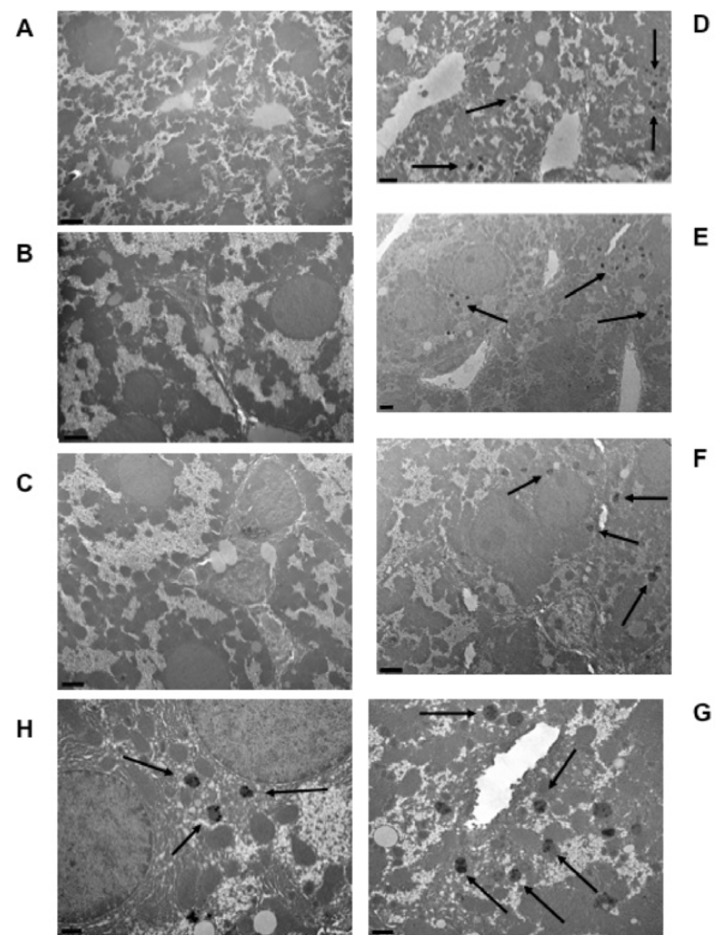
Metal deposits in liver cells of wild-type mice after 7 months of exposure to an aimara-containing diet. Transmission electron microscopy was performed on the liver from control mice (A–C) and aimara-fed mice (D–H). Scale bars appear in the left bottom corners of photos and represent 5 μm (A), 2 μm (B–F) and 1 μm (G and H). Black arrows indicate the metal deposits.

Autometallographic detection allowed us to detect metal deposits within the cytoplasm of hepatic cells. These metal clusters appeared as globular bodies containing little stains denser to electrons than the surrounding part of the globule (Figure 2). These metal corpuscles could only be observed in hepatic cells from mice fed the aimara-containing diet (panels D–H) and were absent in liver cells from those fed the control diet (panels A–C).

## 4. Discussion

The aimara-containing diet selected in this study is aimed to mimic the mercurial contamination of the Wayana population through fish consumption. The aimara diet is enriched in MeHg, whereas the salmon and control diets contain only trace amounts of MeHg. Therefore, as a rule of thumb, any effects specifically observed in mice fed the aimara diet should be attributed to MeHg. Effects observed in mice fed on both the aimara and salmon diets should be attributed to nutrients contained in fish flesh and common to salmon and aimara species.

In a previous article [12], with the same aimara-containing diet and at the same dose, a four-fold up-regulation of *Cox1* and *Mt2* genes in the liver was observed after a one-month exposure. In the present article, and after three and 14 months of exposure, we observed a four- and 14-fold down-regulation of *Cox1* and *Mt2* genes, respectively. This apparent discrepancy might well be due to the levels of accumulated total mercury in the liver which were much higher at three and 14 months, as compared to one month of exposure, with concentrations equal to 67, 77 and 6.7 ng Hg/g, respectively. Thus, the same type of regulation pattern with 10- and 11-times more accumulated mercury cannot be expected.

After three months of exposure, the aimara diet specifically resulted in the brain in the up-regulation of several genes encoding respiratory subunit proteins (*Cox1* and *Cytb*), and superoxide dismutase isoforms (*Sod1* and *Sod2*). This impact vanished after 14 months, suggesting that this organ could adapt or tolerate MeHg with time. The same holds true in the kidney in which the aimara diet-specific effect on the down-regulation of three respiratory genes was obvious after three months, but disappeared after 14 months. In muscles, the reverse was observed since a specific effect of the aimara diet could be observed after 14 months but not after three. The effects of aimara flesh were constant with time in the liver in which all the 15 tested genes were still down-regulated after 14 months. Correspondingly, we observed a decreased respiratory activity in isolated mitochondria from the brain, the kidney and liver after three months of exposure (data not shown). Finally, the aimara-associated MeHg effects were most prominent in the liver, despite the fact that this organ accumulated 13 and six-times less mercury than the kidney after three and 14 months, respectively. Therefore, there is not an obligate relationship between mercury accumulation and effect intensity. This fact was confirmed by the histological impact observed on hepatic cells which area distribution shifted towards the greater values.

The metallothionein protein (MT) quantification shows that in those tissues in which MT concentration increased, MeHg demethylation occurred. This holds true for the kidney after three and seven months of exposure, and in muscles at seven months, in which tissues 40, 100, and 120% increases in MT concentrations were noted, respectively, paralleled by a 40, 70, and 10% of MeHg demethylation. This can be taken as a molecular sign of divalent mercurial contamination, since only Hg^2+^ has been reported yet to induce MT accumulation in contaminated tissues. However, the reciprocal situation does not stand since MeHg demethylation is not necessarily accompanied by a MT concentration increase. This is the case in the liver in which a 30 and 70% MeHg demethylation was noticed after three and seven months of exposure, respectively, with a level of MT similar to control at three months and significantly decreased (36%) at seven months. This peculiar situation can be explained by the 127-times down-regulation of *mt2* gene in liver at three months, a repression still maintained at 14 months. The reason why the *mt2* gene is repressed in a mercury demethylating tissue is unknown, but this illustrates that at low doses of metal contaminant, far below those used in classical studies, unexpected gene response can arise. In addition to this genetic parameter, the decrease of MT level in contaminated liver after seven months of exposure is also probably linked to the formation of insoluble corpuscles of this metal in liver cells (as seen in microscopy), which decreases the proportion of bioavailable inorganic mercury for MTs.

The suppression of the synthesis of the chemokine CCL2 in the corresponding KO mice resulted in important changes in gene expression patterns in the liver and brain. After three months of exposure to aimara-containing diet, eight genes over 10 selected (*Sdhb*, *Cytb*, *Cox1*, *Sod1*, *Sod2*, *Mt2*, *Mdr1a* and *Bax*) were repressed in wild-type mice liver whereas none presented a differential expression in KO *Ccl2*^−/−^ mice. In wild-type mice brain, six genes over 12 selected (*Cytb*, *Cox1*, *Sod1*, *Sod2*, *Mdr1a* and *Bax*) presented a stimulated expression, whereas all of them remained at the basal level of expression in KO *Ccl2*^−/−^ mice. Chemokines were first identified as being responsible for the maturation and trafficking of leukocytes, in particular in inflammatory diseases [26]. Therefore, the results obtained in the liver suggest that the aimara diet-induced repression of genes involved in the mitochondrial respiration (*Sdhb*, *Cytb*, and *Cox1*), in the oxidative stress neutralization (*Sod1* and *Sod2*), and in xenobiotic detoxification (*Mdr1a*), is triggered by an inflammatory process in which intervenes the CCL2 action. In keeping with this, the involvement of CCL2 in molecular mechanisms of hepatic damage has been assessed in mice lacking CCL2 after a carbon tetrachloride (CCl_4_) challenge, since this toxic determines liver injury, inflammation and oxidative stress. In KO *Ccl2*^−/−^ mice, the lack of CCL2 afforded protection from CCl_4_-induced damages and the development of oxidative stress [27]. Several data indicate that many hepatic pathologies are linked to CCL2. For instance, (1) liver fibrosis depends on recruitment of monocytes into the liver and precisely the chemokine receptor CCR2 and its ligand CCL2 participate in regulating monocyte subset infiltration [28,29]; (2) CCL2 induces human hepatoma cell migration and invasion [30]; (3) Obesity activates hepatocyte expression of CCL2 leading to hepatic recruitment of CCR2(+) myeloid cells that promote hepatosteatosis. Reciprocally, reduced hepatic steatosis in obese mice deficient in the CCR2 receptor was observed [31]; (4) inhibition of CCR2 could improve diet-induced obesity and related metabolic disorders, such as insulin resistance and hepatic steatosis, by suppressing inflammation in adipose tissue [32]; (5) hyperhomocysteinemia is a metabolic disorder associated with liver injury and chronic inflammation through induction of CCL2 production in the liver [33]. With such a scheme in mind, one may propose that the CCL2-mediated repression of genes in the liver impedes this organ to mount a proper response against MeHg contamination, and constitutes one of the molecular bases accounting for MeHg toxicity in the liver. The situation in the brain is reversed: instead of a CCL2-based gene repression we observed a CCL2-based gene stimulation. In such a case, in the brain, the lack of CCL2 production would impede the brain in adapting to MeHg toxic effects. In agreement with such a view, we have reported that on the same groups of mice than used in the present study, the aimara-containing diet in the mice cortex provoked a decrease in CCL2 concentrations, a neuronal cell death and a microglial activation. *Ccl2* KO mice that were fed a vegetal control food already presented a decrease in cortical neuronal cell density in comparison with wild-type animals under similar diet conditions showing that the presence of CCL2 is required for normal neuronal survival. Moreover, *Ccl2* KO mice showed a more pronounced cortical neuronal cell death than wild-type mice in response to the aimara diet [16]. Thus, CCL2 would contribute to MeHg toxicity in the liver, and protect against it in the brain.

One may ask about the relevance of MeHg liver effects to clinical or population-based adverse effects. Indeed, most of studies dealing with MeHg focus on neurotoxic outcomes making believe that the liver is not a target for MeHg toxicity. First, it has been reported that deaths from liver cancer, chronic liver disease, and liver cirrhosis were significantly more frequent among registered Minamata disease patients than among the general population of Minamata City [34]. And in rodents, numerous observations showed a MeHg-induced hepatic toxicity. Rats, exposed daily for 4 weeks to 1.0 mg Hg/kg of body weight, were described as having decreased activities in glucose 6-phosphatase, alkaline phosphatase, ATPase and succinic dehydrogenase in the liver [35], along with hepatic mitochondria swelling [36]. In kittens fed daily for 11 months with tuna containing 0.3–0.5 mg Hg/kg, ultrastructural changes were observed in the liver with proliferation of the smooth endoplasmic reticulum and abnormal mitochondria morphology [37]. In rats exposed to 140 mg MeHg/kg/day for 100 days, leukocyte infiltration was observed in the liver [38]. MeHg triggers an oxidative stress in the liver since it has been shown in mice after MeHg exposure, hepatic lipid peroxidation [39], increase in thiobarbituric acid reactive species, decrease in ascorbic acid content [40], and inhibition of the hepatic thioredoxin reductase [41]. In rat liver, after MeHg exposure, a decrease in glutathione level [42], an increased superoxide generation in mitochondria, and increased glutathione peroxidase and decreased superoxide dismutase activities [43] were observed. Oxidative DNA damages in the liver after MeHg exposure have been demonstrated [42,44], and also MeHg-induced hepatic DNA methylation [45]. These published data fit well with our data showing ultrastructural modifications and repression of respiratory genes and superoxide dismutase genes in the liver.

When addressing the question as to whether the mouse is an appropriate model for the mercurial intoxication of the Wayana Amerindians, a good criterion consists in a comparison of the trophic transfer rate of mercury. Dietary MeHg is readily and efficiently absorbed by the human gastrointestinal tract, to a reported level of 95% to 100% [46]. Here, we could calculate a trophic transfer rate of 57% for wild-type mouse, meaning that the impact of aimara diet could be greater in the case of Wayanas Amerindians. This trophic transfer rate is close to that recorded in rats for which the fecal excretion of mercury was about 68% [25].

In the present study, mice were fed an aimara diet representing a daily contamination pressure of 1 μg/kg body weight. This is far below the doses of MeHg given to mice found in the literature. A literature survey of the recent articles dealing with MeHg contamination in mice through diet or drinking water these last 2 years, identifies the commonly-used daily doses which were in decreasing order of concentration: 5.4 [47], 2 [40], 1.4 [48], 1 [49,50], and 0.5 mg MeHg/kg body weight [51]. These concentrations are 5400- to 500-times greater than the contamination pressure we have used in the present study. Finally, it has been found that MeHg could impair motor and cognitive functions in mice daily gavaged or fed with 50 [52] and 10 μg MeHg/kg body weight [53], respectively, equivalent to doses 50 and 10-times higher than in the present study. In the latter work, the mercury content in the brains of adult mice exposed as adults was 63 ng/g, fresh weight (fw), and that in the 3-month old mice exposed prenatally was 28 ng/g, fw [53]. This is three times higher than the value of 10 ng/g, fw (31 ng/g, dw) found in the brains of mice fed the aimara diet in the third experiment of the present study. The reported 90-percentile value of mercury concentration in the cortex of individual Norwegian people without occupational exposure to mercury was found to be 28 ng/g, fw [54], a value 3-times higher than that in the brain of aimara-fed mice in the present study. Therefore, after just three months of feeding with a diet containing 0.15% aimara flesh, mouse brain mercury levels were three-fold below the highest values found in human brains from heavy fish consumers. Swedish people are modest fish consumers and the mercury mean concentrations in the human kidney from the Swedish population are 0.7 μg/g, dw, for women and 0.4 μg/g, dw, for men with an overall distribution range of 0.04–2.1 μg/g, dw [55]. In the kidneys of mice fed 0.15% aimara flesh over three months (experiment 3), the tissue mercury concentration reached a value of 0.91 μg/g, dw, equivalent to mid-range values found in the human kidney from modest fish consumers.

## 5. Conclusions

To conclude, the mercury concentrations found in the tissues of mice fed the aimara diet are in the range of those of humans considered as modest fish consumers, and are therefore fully environmentally relevant. Although MeHg is a neurotoxic compound, we found impacts in the liver after three months of exposure to aimara-containing diet, as illustrated by the repression of eight genes over 10 selected (*Sdhb*, *Cytb*, *Cox1*, *Sod1*, *Sod2*, *Mt2*, *Mdr1a* and *Bax*), and histological alterations for an accumulated mercury concentration as low as 32 ng/g, dw, along with metal deposits within the cytoplasm of hepatic cells.

## Figures and Tables

**Table 1:** The composition of the salmon flesh and diets used. a

Element	Control Diet	Salmon Flesh	Salmon Diet	Aimara Diet
Moisture	10	0.1	10	10
Fat	2.71	42.5	2.77	2.71
Protein	14.38	54.1	14.46	14.46
Fiber	4.65	<0.1	4.65	4.65
Ash	6.00	3.29	6.00	6.00
**Carbohydrates**				
Starch	44.97	0	44.97	44.97
Sugar	4.05	0	4.05	4.05
Pectin	1.52	0	1.52	1.52
Hemicellulose	10.17	0	10.17	10.17
Cellulose	4.32	0	4.32	4.32
Lignin	1.68	0	1.68	1.68
**Fatty acids**				
Saturated fatty acids				
C12:0 Lauric	0.02	0.019	0.02	0.02
C14:0 Myristic	0.14	1.22	0.14	0.14
C16:0 Palmitic	0.31	3.66	0.31	0.31
C18:0 Stearic	0.04	0.814	0.04	0.04
Monounsaturated fatty acids				
C14:1 (ω5) Myristoleic	0.02	0.011	0.02	0.02
C16:1 (ω 7) Palmitoleic	0.09	1.27	0.09	0.09
C18:1 (ω 9) Oleic	0.77	14.032	0.79	0.77
Polyunsaturated fatty acids				
C18:2 (ω 6) Linoleic	0.69	3.853	0.69	0.69
C18:3 (ω 3) Linolenic	0.06	1.53	0.06	0.06
C20:4 (ω 6) Arachidonic	0.13	0.116	0.13	0.13
C20:5 (ω 3) (EPA)	<0.002	1.624	0.002	<0.002
Eicosapentaenoic				
C22:1 (ω 9) Erucic	0.002	0.269	0.002	0.002
C22:5 (ω 3) (DPA)	<0.002	0.795	<0.002	<0.002
Docosapentaenoic				
C22:6 (ω 3) (DHA)	<0.002	2.132	0.003	<0.01
Docosahexaenoic				

a Nutrients and compounds are given as their percentages in the diets.

**Table 2:** The metal composition of the salmon flesh and diets used. a

Element	Control Diet	Salmon Flesh	Salmon Diet	Aimara Diet
Al	41.1	<2	41.1	41.1
As	<0.1	4.6	<0.1	<0.1
Cd	0.064	<0.02	0.064	0.064
Co	0.80	<0.1	0.80	0.80
Cr	0.72	<0.25	0.72	0.72
Cu	7.99	0.71	7.99	7.99
Hg (total)	0.001	0.098	0.001	0.005
Ni	0.39	<0.25	0.39	0.39
Pb	0.165	<0.05	0.165	0.165
Se	0.30	1.1	0.30	0.30
Zn	41.1	12.4	41.1	41.1

a Metals are given in mg.kg^−1^.

**Table 3:** Mercury bioaccumulation in tissues from mice fed aimara-containing diet for 19 months. a

Tissue	3 Months		7 Months		19 Months	
	
	Control	Aimara b	Control	Aimara	Control	Aimara
Hair	35 ± 21	190 ± 10 ^*^	24 ± 2	182 ± 27 ^*^	4 ± 0.3	322 ± 59 ^*^
Kidney	6.0 ± 2.7	298 ± 14 ^*^	6.3 ± 0.3	306 ± 27 ^*^	4.9 ± 0.5	232 ± 42 ^*^
Liver	1.7 ± 2.9	32 ± 6 ^*^	4.6 ± 0.6	32 ± 2 ^*^	1.1 ± 0.1	42 ± 1 ^*^
Muscles	2.4 ± 1.4	19.4 ± 1.0 ^*^	2.1 ± 1.4	21 ± 5 ^*^	6.4 ± 1.3	32 ± 7 ^*^
Brain	<DT	14.8 ± 0.6 ^*^	<DT	12.5 ± 5.2 ^*^	<DT	20.8 ± 0.8 ^*^
Hippocampus	<DT	47 ± 12 ^*^	<DT	17 ± 8 ^*^	ND	ND
Cortex	<DT	20 ± 17 ^*^	ND	ND	ND	ND
Cerebellum	<DT	15 ± 12 ^*^	ND	ND	ND	ND
Spinal cord	<DT	5.1 ± 1.4 ^*^	ND	ND	ND	ND
Lung	<DT	21 ± 4 ^*^	<DT	25 ± 4 ^*^	1.7 ± 0.9	30 ± 21 ^*^
Intestines	<DT	16 ± 1.5 ^*^	<DT	9 ± 1 ^*^	11.8 ± 1.2	21 ± 2 ^*^
Heart	<DT	20 ± 6 ^*^	<DT	13 ± 10 ^*^	7.2 ± 0.6	29 ± 1 ^*^
Spleen	<DT	20 ± 3 ^*^	<DT	<DT	0.8 ± 0.1	18 ± 3 ^*^
Stomach	<DT	15 ± 9 ^*^	<DT	25 ± 3 ^*^	2.2 ± 0.3	23 ± 5 ^*^
Skin	<DT	12 ± 6 ^*^	<DT	24 ± 13 ^*^	ND	ND
Testis	3.9 ± 3.4	19 ± 9 ^*^	1.0 ± 1.0	10 ± 6 ^*^	ND	ND
Bones	<DT	27 ± 26 ^*^	<DT	6 ± 4 ^*^	ND	ND
Blood cells	<DT	10 ± 1 ^*^	<DT	7 ± 4 ^*^	0.5 ± 0.1	11 ± 1.4 ^*^
Feces	<DT	21 ± 7 ^*^	<DT	26 ± 6 ^*^	ND	ND

a Expressed in ng/g (mean ± SD, *n* = 4) on a dry weight basis;

b Aimara: mice were fed a 0.1% aimara flesh-containing diet; <DT: below the detection threshold; ND: not determined;

The asterisk indicates a significant mercury accumulation in tissues of mice fed the aimara diet compared to those fed the control diet, as determined with the Mann-Whitney *U*-test, ^*^ *p* < 0.05.

**Table 4:** Contribution of the methylated form of mercury in tissues from mice fed an aimara-containing diet for 7 months a.

Tissue	3 Months	7 Months
Kidney	61 ± 9 °	29 ± 8 °,^*^
Liver	68 ± 7 °	32 ± 12 °^,*^
Muscles	96 ± 2	89 ± 3 °^,*^
Brain	118 ± 6	111 ± 10

a Values are the contributions of MeHg to the total tissue mercury burden, expressed in percent (*n* = 3, mean ± SD);

The circle indicates a contribution of the methylated form of mercury significantly different from 100% after three or seven months of exposure, as determined with the Mann-Whitney *U*-test, ° *p* < 0.05; The asterisk indicates a significant decrease of the methylated form of mercury between three or seven months of exposure, as determined with the Mann-Whitney *U*-test, ^*^ *p* < 0.05.

**Table 5:** Mercury bioaccumulation in tissues from mice fed fish-containing diets for 14 months a.

Tissue	Time (Months)	Control	Salmon b	Aimara c
Hair	3	3.4 ± 1.2	6.5 ± 2	242 ± 11 ^*^
	14	33 ± 26	30 ± 8	733 ± 201 ^*^
Kidney	3	12 ± 3	16 ± 2	359 ± 200 ^*^
	14	7 ± 2	21 ± 2 °	511 ± 1034 ^*^
Liver	3	4.8 ± 0.4	4.5 ± 1.2	34 ± 18 ^*^
	14	4 ± 2.6	7.7 ± 1.4	77 ± 11 ^*^
Muscles	3	6 ± 2	6 ± 2	34 ± 18 ^*^
	14	8 ± 8	13 ± 2	53 ± 11 ^*^
Brain	3	9 ± 2	9 ± 2	23 ± 5 ^*^
Hippocampus	14	4 ± 5	11 ± 7	46 ± 4 ^*^
Cortex	14	1.5 ± 0.4	11 ± 7	35 ± 3 ^*^
Striatum	14	3 ± 3	11 ± 7	56 ± 11 ^*^
Eyes	14	3 ± 2	18 ± 5 °	61 ± 24 ^*^
Lung	14	8 ± 4	9 ± 2	74 ± 7 ^*^
Intestines	14	12 ± 11	4.1 ± 0.3	48 ± 9 ^*^
Heart	14	4 ± 3	7 ± 4	43 ± 9 ^*^
Spleen	14	9 ± 3	18 ± 11	45 ± 3 ^*^
Skin	14	18 ± 12	7 ± 4	24 ± 5 ^*^
Blood	14	6.4 ± 0.1	7.2 ± 0.1	27.7 ± 2.5 ^*^
Feces	14	6.5 ± 0.3	6.5 ± 1.1	34.8 ± 1.4 ^*^

a Expressed in ng/g ± SD (*n* = 4) on a dry weight basis;

b Salmon: mice were fed a 0.15% salmon flesh-containing diet;

c Aimara: mice were fed a 0.15% aimara flesh-containing diet;

The asterisk indicates a significant mercury accumulation in tissues of mice fed the aimara diet compared to those fed both the control and salmon diets, as determined with the Mann-Whitney *U*-test, ^*^ *p* < 0.05; The circle indicates a significant mercury accumulation in tissues of mice fed the salmon diet compared to those fed the control diet, as determined with the Mann-Whitney *U*-test, ° *p* < 0.05.

**Table 6:** Mercury bioaccumulation in tissues from mice fed fish-containing diets for three months. a

Tissue	C57Black6 Wild-Type (*Ccl2*^+/+^)			C57Black6 KO (*Ccl2*^−/−^)	
		
	Control	Salmon b	Aimara c	Control	Aimara c
Hair	62 ± 35	24 ± 15	470 ± 137 ^*^	28 ± 15	475 ± 190 ^*^
Kidney	63 ± 64	43 ± 12	907 ± 142 ^*^	37 ± 10	751 ± 239 ^*^
Liver	5 ± 4	4.4 ± 0.8	67 ± 7 ^*^	6 ± 3	100 ± 47 ^*^
Muscles	2.6 ± 1.3	3.6 ± 2.0	56 ± 4 ^*^	4.5 ± 3.5	78 ± 27 ^*^
Brain	2.6 ± 0.7	2.7 ± 0.6	31 ± 14 ^*^	2.9 ± 1.5	32 ± 9 ^*^
Eyes	8 ± 6	3 ± 2	65 ± 38 ^*^	5 ± 4	70 ± 19 ^*^
Blood	0.9 ± 0.7	1.9 ± 0.2	32 ± 5 ^*^	1.0 ± 0.9	37 ± 15 ^*^
Feces	2.4 ± 1.3	2.2 ± 0.9	19 ± 3 ^*^	3.3 ± 0.1	17 ± 6 ^*^

a Expressed in ng/g ± SD (*n* = 4) on a dry weight basis;

b Salmon: mice were fed a 0.15% salmon flesh-containing diet;

c Aimara: mice were fed a 0.15% aimara flesh-containing diet;

The asterisk indicates a significant mercury accumulation in tissues of mice fed the aimara diet compared to those fed both the control and salmon diets, as determined with the Mann-Whitney *U*-test, ^*^ *p* < 0.05.

**Table 7:** Metallothionein concentrations in tissues from mice fed aimara-containing diet for 7 months a.

Tissue	3 Months		7 Months	
		
	Control	Aimara	Control	Aimara
Kidney	11.3 ± 0.6	15.8 ± 1.3 ^*^	4.0 ± 0.1	8.0 ± 1.2 ^*^
Liver	7.4 ± 1.4	10.8 ± 1.4	14.6 ± 2.5	9.3 ± 0.4 ^*^
Muscles	4.6 ± 0.6	3.9 ± 0.9	2.5 ± 0.2	5.5 ± 0.2 ^*^
Brain	47.2 ± 2.2	53.0 ± 2.8	54.1 ± 8.1	50.0 ± 8.4

a Values are the number of mercury binding sites presented by metallothioneins, expressed in nmol bound Hg/g fresh weight (mean ± SEM, *n* = 3);

The asterisk indicates a significant different metallothionein concentration in tissues of the mice fed the aimara diet compared to those fed the control diet, as determined with the Mann-Whitney *U*-test, ^*^ *p* < 0.05.

**Table 8:** Gene expression in tissues from wild-type mice fed fish-containing diets for three months a.

Tissue and Gene	Control	Salmon	Aimara
**Liver**			

*Ndusf8*	99 ± 67	111 ± 65	16.5 ± 13
*Sdhb*	303 ± 113	^§^ 750 ± 129	^*^ 192 ± 104
*Cytb*	10,969 ± 4,066	^§^ 43,691 ± 454	^*^ 9,043 ± 4,757
*Cox1*	1,256 ± 328	^§^ 6,037 ± 1,397	^*^ 883 ± 442
*Atp5a1*	180 ± 96	460 ± 244	88 ± 48
*Sod1*	2,450 ± 1,402	2,624 ± 272	^*^ 606 ± 341
*Sod2*	167 ± 79	180 ± 18	^*^ 33.3 ± 16.5
*Mt2*	10,155 ± 7,236	1,203 ± 382	^*^ 80 ± 41
*Mdr1a*	1.2 ± 0.5	1.5 ± 0.4	^*^ 0.4 ± 0.1
*Bax*	11.7 ± 5.3	12.3 ± 2.3	^*^ 3.6 ± 1.9

**Kidney**			

*Mt-Nd4*	5,689 ± 2,338	4,541 ± 4,148	° 761 ± 323
*Ndusf8*	11.8 ± 4.5	6.8 ± 2.8	3.0 ± 0.4
*Sdhb*	27.4 ± 12.2	19.1 ± 12.4	9.2 ± 2.7
*Cytb*	2,746 ± 1,373	1,388 ± 856	1,098 ± 409
*Cox1*	881 ± 305	656 ± 374	769 ± 423
*Cox4*	234 ± 70	231 ± 132	° 29.9 ± 8.0
*Atp5a1*	22.9 ± 10.7	17.2 ± 7.3	26.0 ± 18.7
*Atp6*	15,412 ± 4,619	10,609 ± 7,533	° 1,126 ± 250
*Sod1*	44.0 ± 26.8	12.9 ± 4.8	32 ± 12
*Sod2*	1.0 ± 5.2	4.1 ± 2.8	3.3 ± 1.1
*Mt2*	9.3 ± 4.9	4.3 ± 1.6	6.2 ± 2.6
*Mdr1a*	0.07 ± 0.02	0.11 ± 0.07	0.13 ± 0.06
*Bax*	1.3 ± 0.5	1.1 ± 0.4	1.7 ± 0.9

**Muscles**			

*Ndusf8*	85 ± 12	^§^ 42 ± 14	° 38 ± 17
*Sdhb*	394 ± 64	^§^ 211 ± 32	° 168 ± 63
*Cytb*	14,793 ± 2,657	10,944 ± 2,842	6,933 ± 2,600
*Cox1*	3,465 ± 504	5,150 ± 1,400	3,239 ± 1,149
*Atp5a1*	357 ± 69	151 ± 45	139 ± 65
*Sod1*	61.5 ± 5.6	^§^ 35.3 ± 4.7	° 30.3 ± 8.5
*Sod2*	50.8 ± 3.4	^§^ 27.8 ± 7.4	° 27.3 ± 10.5
*Mt2*	71 ± 61	49.5 ± 43.7	5.9 ± 4.0
*Mdr1a*	0.45 ± 0.17	0.26 ± 0.05	0.26 ± 0.12
*Bax*	2.2 ± 0.3	^§^ 1.1 ± 0.3	° 0.9 ± 0.3

**Brain**			

*Ndusf8*	64 ± 14	44 ± 11	^*^ 88 ± 12
*Sdhb*	54 ± 18	61 ± 19	91 ± 13
*Cytb*	5,348 ± 888	4,830 ± 1,275	^*^ ° 7,919 ± 499
*Cox1*	4,519 ± 1,030	7,287 ± 2,324	° 11,851 ± 1,684
*Atp5a1*	151 ± 36	152 ± 36	285 ± 54
*Sod1*	28.3 ± 7.9	45.5 ± 13.9	° 56.7 ± 4.5
*Sod2*	14.6 ± 2.9	22.0 ± 6.0	° 37.4 ± 4.7
*Mt2*	195 ± 52	215 ± 112	333 ± 68
*Mdr1a*	2.2 ± 0.5	3.4 ± 1.4	° 4.9 ± 0.6
*Bax*	4.9 ± 0.9	7.70 ± 3.0	° 11.4 ± 1.9
*Cd11b*	0.5 ± 0.2	^§^ 2.1 ± 0.6	° 5.7 ± 1.7
*Mcp1*	0.06 ± 0.03	^§^ 7.3 ± 1.5	° 7.9 ± 1.5
*Gfap*	24 ± 9	109 ± 58	° 151 ± 80

a Relative gene expressions (mean ± SEM, *n* = 5). *b-actin* was the reference gene;

Asterisks and circles indicate a significant differential gene expression in tissues of mice fed the aimara diet compared to those fed the salmon and the control diets, respectively, as determined with the Mann-Whitney *U*-test, ^*^ and ° *p* < 0.05; The symbol ^§^ indicates a significant differential gene expression in tissues of mice fed the salmon diet compared to those fed the control diet, as determined with the Mann-Whitney *U*-test, ^§^ *p* < 0.05.

**Table 9:** Gene expression in tissues from wild-type mice fed fish-containing diets for 14 months a.

Tissue and Gene	Control	Salmon	Aimara
**Liver**			

*Mt-Nd4*	63,087 ± 18,729	39,106 ± 13,620	° ^*^ 4,928 ± 1,809
*Ndusf8*	1,699 ± 536	805 ± 271	° ^*^ 157 ± 74
*Sdhb*	1,753 ± 559	1,676 ± 722	° ^*^ 189 ± 80
*Uqcrc2*	537 ± 183	850 ± 287	° ^*^ 105 ± 45
*Cytb*	224,728 ± 77,406	185,711 ± 83,232	° ^*^ 16,068 ± 6,337
*Cox1*	52,550 ± 25,693	43,713 ± 16,252	° ^*^ 6,172 ± 2,987
*Cox4*	11,340 ± 3,288	18,505 ± 4,388	° ^*^ 1,388 ± 638
*Atp5a1*	1,060 ± 394	919 ± 388	° ^*^ 110 ± 46
*Atp6*	395,164 ± 176,039	473,579 ± 168,995	° ^*^ 24,540 ± 7,087
*Gsta4*	919 ± 291	408 ± 150	° ^*^ 39 ± 18
*Sod1*	8,160 ± 5,004	6,804 ± 1,734	^*^ 668 ± 332
*Sod2*	95 ± 54	195 ± 99	^*^ 20 ± 10
*Mt2*	519 ± 426	667 ± 154	^*^ 48 ± 36
*Hsp25*	114 ± 40	161 ± 70	° ^*^ 21 ± 12
*Mdr1a*	0.65 ± 0.12	2.0 ± 0.5	° ^*^ 0.2 ± 0.1

**Kidney**			

*Mt-Nd4*	167,243 ± 94,180	138,080 ± 76674	170,635 ± 74,914
*Ndusf8*	8,540 ± 3,332	^§^ 1,267 ± 632	° 886 ± 340
*Sdhb*	721,117 ± 418,327	^§^ 3,005 ± 1,399	° 1,568 ± 637
*Uqcrc2*	10,110 ± 4,914	1,493 ± 691	795 ± 278
*Cytb*	620,950 ± 317,570	573,561 ± 247,300	383,586 ± 138,034
*Cox1*	297,861 ± 127,298	340,759 ± 239,810	329,034 ± 122,757
*Cox4*	51,011 ± 23,243	25,720 ± 12,488	11,068 ± 4,322
*Atp5a1*	24,045 ± 18,904	2,216 ± 1,137	1,183 ± 526
*Atp6*	1,378,186 ± 689,089	1,279,839 ± 469,065	728,398 ± 256,796
*Gsta4*	3,855 ± 2,893	^§^ 197 ± 70	° 69 ± 29
*Sod1*	16,498 ± 7,933	4,689 ± 2,592	2,200 ± 630
*Sod2*	5,916 ± 2,947	^§^ 331 ± 163	° 192 ± 77
*Mt2*	7,000 ± 4,756	^§^ 418 ± 208	° 181 ± 73
*Hsp25*	25,833 ± 23,725	^§^ 64 ± 30	° 30 ± 15
*Mdr1a*	218,236 ± 134,938	^§^ 10 ± 4	° 4 ± 2

**Muscles**			

*Mt-Nd4*	66,678 ± 18,329	140,812 ± 26,814	° ^*^ 12,603 ± 5,773
*Ndusf8*	1,891 ± 200	2,985 ± 938	° ^*^ 178 ± 52
*Sdhb*	4,701 ± 1,311	6,767 ± 1,556	° ^*^ 1,061 ± 456
*Uqcrc2*	1,361 ± 344	2,428 ± 622	° ^*^ 271 ± 62
*Cytb*	143,541 ± 64,704	316,516 ± 56,361	^*^ 33,135 ± 7,360
*Cox1*	84,593 ± 6,890	224,342 ± 133,120	° ^*^ 21,479 ± 4,182
*Cox4*	12,371 ± 2,698	16,840 ± 4,062	° ^*^ 2,605 ± 509
*Atp5a1*	6,847 ± 1,689	7,953 ± 2,499	° ^*^ 316 ± 145
*Atp6*	491,758 ± 90,400	923,159 ± 205,328	° ^*^ 162,612 ± 72,845
*Gsta4*	795 ± 663	135 ± 40	^*^ 26 ± 18
*Sod1*	3,118 ± 2,070	1,691 ± 235	° ^*^ 243 ± 77
*Sod2*	402 ± 134	583 ± 194	° ^*^ 76 ± 33
*Mt2*	570 ± 451	2,536 ± 1,702	168 ± 111
*Hsp25*	1,362 ± 709	1,484 ± 385	344 ± 214
*Mdr1a*	6.1 ± 2.8	7.2 ± 3.0	° ^*^ 0.27 ± 0.11

**Brain**			

*Mt-Nd4*	55,293 ± 7,817	65,745 ± 30,313	40,594 ± 26,017
*Ndusf8*	1,638 ± 133	1,299 ± 293	1,419 ± 538
*Sdhb*	2,908 ± 339	2,379 ± 685	2,473 ± 1,135
*Uqcrc2*	1,507 ± 167	1,031 ± 318	1,171 ± 479
*Cytb*	150,447 ± 27,563	179,445 ± 61,126	183,610 ± 82,933
*Cox1*	164,349 ± 20,662	145,897 ± 31,324	145,860 ± 51,140
*Cox4*	14,588 ± 1,644	16,703 ± 4,705	14,422 ± 7,865
*Atp5a1*	7,684 ± 699	^§^ 4,411 ± 877	5,969 ± 3,046
*Atp6*	412,008 ± 59,859	364,127 ± 112,665	436,034 ± 202,755
*Gsta4*	469 ± 66	353± 94	348 ± 114
*Sod1*	2,143 ± 162	1,812 ± 471	2,677 ± 1,560
*Sod2*	426 ± 38	299 ± 67	215 ± 114
*Mt2*	2,608 ± 393	3,390 ± 939	2,850 ± 1,038
*Hsp25*	165 ± 25	^§^ 83 ± 14	118 ± 71
*Mdr1a*	110 ± 6	^§^ 47 ± 7	66 ± 40

a Relative gene expressions (mean ± SEM, *n* = 5). *b-actin* was the reference gene;

Asterisks and circles indicate a significant differential gene expression in tissues of mice fed the aimara diet compared to those fed the salmon and the control diets, respectively, as determined with the Mann-Whitney U-test, ^*^ and ° *p* < 0.05; The symbol ^§^ indicates a significant differential gene expression in tissues of mice fed the salmon diet compared to those fed the control diet, as determined with the Mann-Whitney U-test, ^§^ *p* < 0.05.

**Table 10:** Differential expression in tissues of mice fed the aimara-containing diet after 3 or 14 months exposure. a

Gene	Liver		Kidney		Muscles		Brain	
				
	3	14	3	14	3	14	3	14
*Mt-Nd4*	x	0.13	0.13	NS	x	0.2	x	NS
*Ndusf8*	NS	0.2	NS	NS	NS	0.09	2	NS
*Sdhb*	0.26	0.11	NS	NS	NS	0.2	NS	NS
*Cytb*	0.2	0.09	NS	NS	NS	0.2	1.5	NS
*Uqcrc2*	x	0.12	x	NS	x	0.2	x	NS
*Cox1*	0.15	0.14	NS	NS	NS	0.25	2.6	NS
*Cox4*	x	0.07	0.13	NS	x	0.2	x	NS
*Atp5a1*	NS	0.12	NS	NS	NS	0.05	NS	NS
*Atp6*	x	0.05	0.07	NS	x	0.3	x	NS
*Sod1*	0.23	0.1	NS	NS	NS	0.08	2	NS
*Sod2*	0.18	0.1	NS	NS	NS	0.2	2.6	NS
*Gsta4*	x	0.09	x	NS	x	0.2	x	NS
*Hsp25*	x	0.13	x	NS	x	NS	x	NS
*Mt2*	0.07	0.07	NS	NS	NS	NS	NS	NS
*Mdr1a*	0.27	0.1	NS	NS	NS	0.04	2.2	NS
*Bax*	0.29	x	NS	NS	NS	x	2.3	x

a Only the significant values due to aimara flesh are indicated. When a differential expression as compared to control was observed for both fish-containing diet, it was not recorded in that table;

NS: non-significant; x: not tested.

**Table 11:** Gene expression in tissues from KO CCL2 mice fed a fish-containing diet for three months a.

Tissue and Gene	Control	Aimara	Ratio b
**Liver**			

*Ndusf8*	35 ± 12	228 ± 188	6.6
*Sdhb*	957 ± 413	1,715 ± 1,282	1.8
*Cytb*	38,722 ± 15,233	44,116 ± 33,819	1.1
*Cox1*	2,979 ± 943	3,017 ± 1,465	1.0
*Atp5a1*	192 ± 76	1,171 ± 1,038	6.1
*Sod1*	1,834 ± 702	6,915 ± 5,940	3.8
*Sod2*	149 ± 67	612 ± 478	4.1
*Mt2*	433 ± 362	77 ± 31	0.2
*Mdr1a*	0.6 ± 0.2	1.0 ± 0.7	1.7
*Bax*	12.7 ± 2.2	22.0 ± 14.2	1.7

**Kidney**			

*Mt-Nd4*	884 ± 426	302 ± 172	0.3
*Ndusf8*	5.2 ± 3.3	5.4 ± 1.9	1.0
*Sdhb*	11.3 ± 5.5	8.4 ± 2.7	0.75
*Cytb*	920 ± 399	1,022 ± 146	1.1
*Cox1*	680 ± 320	989 ± 334	1.4
*Cox4*	51 ± 33	28 ± 19	0.5
*Atp5a1*	18 ± 10	171 ± 140	9.6
*Atp6*	2,677 ± 1,605	863 ± 667	0.3
*Sod1*	19 ± 10	14 ± 5	0.7
*Sod2*	3.5 ± 1.9	3.3 ± 1.2	0.9
*Mt2*	3.3 ± 0.8	2.9 ± 0.1	0.9
*Mdr1a*	0.3 ± 0.1	0.17 ± 0.04	0.6
*Bax*	1.2 ± 0.3	2.0 ± 0.3	1.7

**Muscles**			

*Ndusf8*	17.6 ± 12.0	17.4 ± 7.2	1.0
*Sdhb*	84 ± 37	75 ± 21	0.9
*Cytb*	4,203 ± 2,188	5,630 ± 802	1.3
*Cox1*	2,195 ± 842	1,967 ± 715	0.9
*Atp5a1*	75 ± 54	29 ± 18	0.4
*Sod1*	16.3 ± 8.6	11.2 ± 0.9	0.7
*Sod2*	12.4 ± 6.2	9.1 ± 2.8	0.70
*Mt2*	0.76 ± 0.05	1.36 ± 0.3	1.8
*Mdr1a*	0.12 ± 0.07	0.07 ± 0.04	0.6
*Bax*	0.4 ± 0.3	0.4 ± 0.1	1.0

**Brain**			

*Ndusf8*	59 ± 2	^*^ 36 ± 5	^*^ 0.6
*Sdhb*	64 ± 15	58 ± 12	0.9
*Cytb*	5,544 ± 315	3,892 ± 1,086	0.7
*Cox1*	7,826 ± 843	6,003 ± 79	0.77
*Atp5a1*	200 ± 30	138 ± 26	0.7
*Sod1*	44 ± 8	44 ± 14	1.0
*Sod2*	24 ± 2	18.5 ± 3	0.76
*Mt2*	144 ± 35	114 ± 20	0.8
*Mdr1a*	3.2 ± 0.1	3.6 ± 0.5	1.1
*Bax*	7.6 ± 1.0	7.3 ± 1.2	1.0
*Cd11b*	4.4 ± 0.6	12.4 ± 8.6	2.8
*Gfap*	97 ± 43	136 ± 91	1.4

a Relative gene expressions (mean ± SEM, *n* = 5). *b-actin* was the reference gene;

b The ratio represents the differential expression of genes from aimara-fed mice as compared to control;

The asterisk indicates a significant differential gene expression in the brains of mice fed the aimara diet compared to those fed the control diet, as determined with the Mann-Whitney *U*-test, ^*^ *p* < 0.05.

**Table 12:** Distribution of hepatic cell areas in mice fed aimara-containing diet for 7 months.

Area Class (μm^2^)	Occurrence of Cells (%)	
	
	Control a	Aimara a
100–200	2.4	1.1
200–300	18.8	10.6
300–400	41.2	25.7
400–500	24.7	26.0
500–600	7.8	21.2
600–700	4.7	9.2
700–800	0.4	4.2
800–900	0	1.4
900–1000	0	0.6

a The numbers of observed hepatic cells were 255 and 358 for control and contaminated livers, respectively.
